# Expression and Function of Nicotinic Acetylcholine Receptors in Induced Regulatory T Cells

**DOI:** 10.3390/ijms23031779

**Published:** 2022-02-04

**Authors:** Yuichiro Nakata, Kento Miura, Norimasa Yamasaki, Sawako Ogata, Shuka Miura, Naohisa Hosomi, Osamu Kaminuma

**Affiliations:** 1 Sylvester Comprehensive Cancer Center, Miami, FL 33136, USA; yxn153@med.miami.edu; 2 Department of Disease Model, Research Institute of Radiation Biology and Medicine, Hiroshima University, Hiroshima 734-8553, Japan; kmiura@hiroshima-u.ac.jp (K.M.); hlnkrcf@hiroshima-u.ac.jp (N.Y.); ogata3@hiroshima-u.ac.jp (S.O.); shu0314@hiroshima-u.ac.jp (S.M.); nhosomi@hiroshima-u.ac.jp (N.H.)

**Keywords:** acetylcholine receptors, histone modification, immune regulation, nicotine, T cell

## Abstract

A contribution of the cholinergic system to immune cell function has been suggested, though the role of nicotine and its receptors in T cells, especially regulatory T (Treg) cells, is unclear. We herein investigated the expression and function of nicotinic acetylcholine receptors (nAChRs) in murine-induced Treg (iTreg) cells. Upon differentiation of naive BALB/c T cells into iTreg cells and other T-cell subsets, the effect of nicotine on cytokine production and proliferation of iTreg cells was examined. The expression of nAChRs and its regulatory mechanisms were comparatively analyzed among T-cell subsets. Stimulation-induced transforming growth factor-β1 (TGF-β1) production of iTreg cells was suppressed by nicotine, whereas interleukin (IL)-10 production and proliferation was not affected. α2-, α5-, α9-, and β2-nAChRs were differentially expressed in naive, Th1, Th2, Th9, Th17, and iTreg cells. Among these cell types, the α9-nAChR was particularly upregulated in iTreg cells via its gene promoter, but not through tri-methylation at the 4th lysine residue of the histone H3-dependent mechanisms. We conclude that the immunoregulatory role of Treg cells is modified by the cholinergic system, probably through the characteristic expression of nAChRs.

## 1. Introduction

Cholinergic neurotransmitters and their corresponding nicotinic acetylcholine receptors (nAChRs) play pivotal roles in not only transmitting neuronal signals but also regulating a variety of biological events. The nAChRs function in various cells by forming homo- or hetero-pentamers constructed of combinations drawn from ~17 subunits (α1-10, β1-4, γ, δ, and ε) [1,2]. The recent identification of nAChRs in immune cells has led us to focus on their roles in neuroimmune interactions [3,4]. 

Since cigarette smoke delivers a large amount of nicotine to the respiratory system, the effect of nicotine on airway immune diseases has been investigated. Allergic airway inflammation evoked in murine models of bronchial asthma was suppressed by the treatment with nicotine [5] or 1,1-dimethyl-4-phenylpiperazinium, a synthetic nAChR ligand [6].

We have demonstrated that antigen-induced airway inflammation developed in immunized mice is largely dependent on CD4^+^ T cells [7]. Naive T cells differentiate into various helper T (Th) cell subsets, depending on their surrounding environment [8], including Th2 cells that produce IL-4 and IL-5, essential interleukins for IgE production and eosinophil activation, respectively, and Th1, Th9, and Th17 cells, which have the potential to induce allergic airway inflammation in the absence of IgE/mast cell-dependent responses [9,10]. T-cell subset-dependent inflammation could be induced in other tissues, including intestines [11]. Interestingly, a murine model of Th2-mediated colitis was attenuated, whereas that of Th1/Th17-favored colitis was augmented, following nicotine treatment [12].

Evidence to date suggests that nAChR expression and function differ among T-cell subsets. In addition to Th subsets, regulatory T (Treg) cells play a pivotal role in immune regulation. Parallel to the maturation of Th subsets, naive CD4^+^ T cells can differentiate into Treg cells in peripheral tissues under TGF-β-exposed conditions [13]. Besides natural Treg cells, which are developed in the thymus, peripherally differentiated Treg cells are recognized as induced Treg (iTreg) cells.

A contribution of the cholinergic system to T-cell subset differentiation and function has been reported previously. For example, mecamylamine, a non-selective nAChR inhibitor, suppressed cytokine production and proliferation of T cells [14]. Nicotinic stimulation to CD4^+^CD62L^+^ T cells upregulates and downregulates IFN-γ and IL-17 production, respectively, whereas muscarinic stimulation enhances IL-10 and IL-17 but suppresses IFN-γ secretion [15]. Although the possible contribution of α7-nAChR to the development of T-cell subsets was demonstrated [16], the participation of nicotine and its receptors in Treg cells has not been fully elucidated. In this study, we investigated the effect of nicotine on iTreg cells, along with the expression patterns and mechanisms regulating nAChR expression, in comparison with other T-cell subsets.

## 2. Results

### 2.1. Effect of Nicotine on iTreg Cells

**Figure 1:**
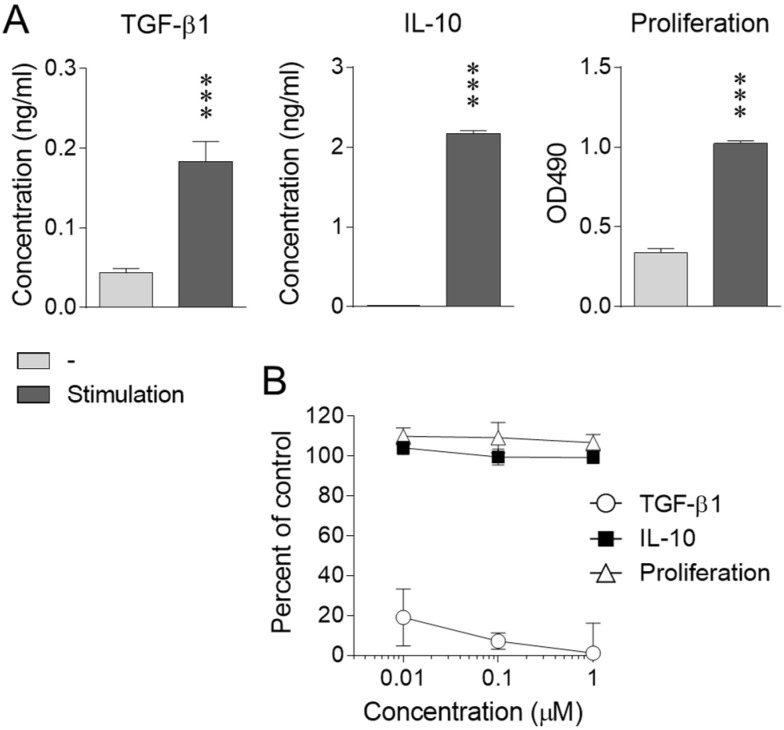
Effect of nicotine on cytokine production and proliferation of iTreg cells. Differentiated iTreg cells were stimulated through CD3/CD28 in the absence (**A**) and presence (**B**) of nicotine. Then, TGF-β1 and IL-10 production and proliferation were assessed. Data are expressed as means ± SD for cytokine concentrations in the culture supernatants, OD490 values elicited by the proliferation assay reagent (**A**), and percent of cytokine production and proliferation relative to stimulated controls (**B**) (*n* = 3). *** *p* < 0.001, compared with the unstimulated control (Student’s *t*-test).

Successful differentiation of iTreg cells and other T-cell subsets has been described elsewhere [10,11,17]. The effect of nicotine on iTreg function was investigated by evaluating the proliferation and expression of characteristic cytokines. Upon activation, iTreg cells produced significant amounts of TGF-β1 and IL-10 and displayed a substantial proliferative response (Figure 1A). Clear and dose-dependent suppression of TGF-β1 production was induced by nicotine, whereas no obvious effect was observed on IL-10 production or proliferation within the same dose range (Figure 1B). 

### 2.2. Expression of nAChRs on T Cell Subsets

**Figure 2:**
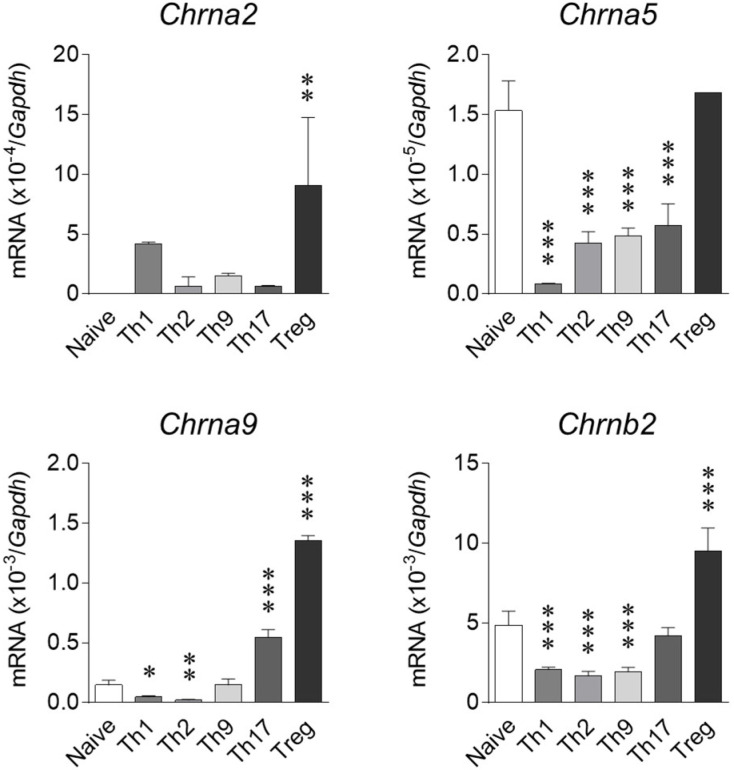
Expression of nAChRs in T-cell subsets. mRNA expression of nAChRs in T-cell subsets and naive CD4^+^ T cells were determined as described in Materials and Methods. Data are expressed as means ± SD of relative mRNA abundance normalized to *Gapdh* expression (*n* = 2–4). * *p* < 0.05, ** *p* < 0.01, *** *p* < 0.001, relative to naive CD4^+^ T cells (Dunnett’s test).

As nicotine exerted a striking effect on iTreg cells, their nAChR expression pattern was analyzed relative to other T-cell subsets and naive CD4^+^ T cells. As shown in Figure 2, *Chrna2* was undetectable in naive cells and upregulated by subset differentiation, particularly into iTreg cells. *Chrna5* substantially expressed in naive cells was downregulated upon differentiation into Th1, Th2, Th9, or Th17 cells. *Chrna9* expression was diminished following Th1 and Th2 differentiation but was enhanced in Th17 and iTreg cells. *Chrnb2* was downregulated in Th1, Th2, and Th9 cells and augmented by iTreg polarization (Figure 2). The other nAChRs, including *Chrna3*, *Chrna4*, *Chrna6*, *Chrna7*, *Chrna10*, *Chrnb3*, and *Chrnb4*, were not detectable in any T-cell population employed in this study (data not shown).

### 2.3. Mechanisms Regulating nAChR9 Expression in iTreg Cells

**Figure 3:**
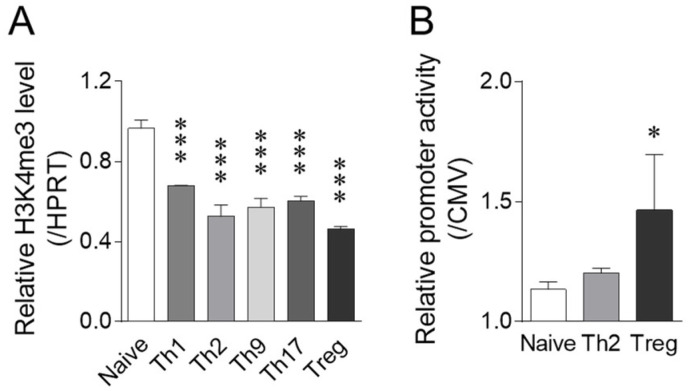
*Chrna9* gene H3K4me level and promoter activity. H3K4me3 level (**A**) and promoter activity (**B**) in T-cell subsets and naive CD4^+^ T cells were determined as described in Materials and Methods. Data are expressed as means ± SD normalized to H3K4me3 levels in the *Hprt* gene (**A**) and to CMV promoter activity (**B**), respectively (*n* = 3). * *p* < 0.05, *** *p* < 0.001, compared with naive CD4^+^ T cells (Dunnett’s test).

Following iTreg differentiation, significant increases in *Chrna2* and *Chrna9* expression were observed at the mRNA level. As a detailed analysis of *Chrna2* gene expression has been performed by Bessis et al. [18], we examined regulatory mechanisms underlying nAChR9 expression in iTreg cells, including histone modification and promoter activity. Tri-methylation at H3K4, which is closely related to the gene transcription activation [19], was comparatively examined in T-cell subsets. The H3K4me3 level was slightly and similarly downregulated following differentiation into iTreg cells and other T-cell subsets (Figure 3A). By contrast, *Chrna9* gene promoter activity was significantly elevated in iTreg cells, but not Th2 cells, relative to naive CD4^+^ T cells (Figure 3B). These findings suggest that the strong *Chrna9* gene upregulation in iTreg cells was mainly caused by promoter activation rather than chromatin modification, at least at the H3K4me3 level.

## 3. Discussion

Our present study clearly demonstrated that TGF-β1 production of iTreg cells was suppressed by nicotine, though IL-10 production and proliferation were not. TGF-β1 is a multifunctional cytokine produced by various cells. In particular, the production of TGF-β1, which induces peripheral T-cell tolerance, is a characteristic feature of Treg cells [13]. The production of biologically active TGF-β1 involves multiple post-translational steps, including processing, dimerization, secretion with or without association with latent-TGF-β-binding protein, and activation through proteolysis and conformational shift [13]. However, the ELISA we employed in this study detected any form of secreted TGF-β1, suggesting that nicotine targets a relatively early stage in its processing.

We have demonstrated that IL-4 production and the proliferation of Th2 cells were mildly suppressed by nicotine [20]. IL-10, another characteristic Treg cytokine, is also produced by Th2 cells, although its molecular regulation differs among T-cell subsets [21]. To investigate reasons for the differences in nicotine’s effect on T-cell subset functions observed in our current and previous studies, systematic analyses, including a detailed time-course study, of genetic, epigenetic, post-transcriptional, and post-translational mechanisms regulating the production of multiple cytokines may be desirable.

nAChRs expression was dynamically affected by T-cell subset differentiation. This pattern partly differs from previous findings demonstrated by Qian et al. [15]. Consistent with our findings, Qian et al. noted decreases in α9- and β2-nAChRs following polarization toward Th2 and Th1/Th17 cells, respectively. However, in addition to α9-nAChR downregulation in Th17 cells, they showed the expression of α5-, α10, and β4-nAChRs but not α2-nAChR in some T-cell subsets [15]. Among α3-, α5-, α7-, α9-, and α10-nAChRs, which have been reported to express in human T cells [22], a functional role of α7-nAChR in the nicotine-induced Ca2^+^ response was demonstrated [23]. While the reason for this discrepancy is unclear, the expression of nAChRs appears to be affected by the corresponding ligands [24,25], which are potentially produced by their proprietor T cells [4,26]. Such complicated feedback systems may cause the dynamic modulation of nAChR expression patterns in various T-cell fates.

It is intriguing that α9-nAChR expression was oppositely modulated following differentiation into Th1 and Th2 cells compared to Th17 and iTreg cells. A contribution of α9-nAChR to immune responses was demonstrated by gene modification. In vitro overexpression and knockdown experiments revealed α9-nAChR involvement in nicotine-induced PD-ligand 1 expression in melanoma cells [27]. Disease severity in an experimental autoimmune encephalomyelitis (EAE) model was suppressed and exacerbated in α9- and β2-nAChR knockout mice, respectively [28]. Additional disease attenuation was observed in α9/α10-nAChR double-knockout mice [29]. α9-nAChR forms a homo-pentamer or hetero-pentamer with α10-nAChR [2], suggesting that the α9/α10 hetero-pentamer plays a role in immune regulation. However, α10-nAChR was not detected in any of the T cells used in this study. At least in iTreg cells, the α9-nAChR homo-pentamer might participate in mediating the effect of nicotine. On the other hand, α2-, α5-, and β2-nAChRs, which were also detected in this study, potentially compose hetero-pentamers [2]. Since an immunological phenotype was observed in β2-nAChR knock-out mice [28], a detailed examination of the cell surface expression and functional role of these subunits in each T-cell subset may be needed.

As various genetic and epigenetic mechanisms are implicated in gene transcription, we elucidated that α9-nAChR expression is regulated at the promoter activation level. Tai et al. reported that the α9-nAChR was upregulated by nicotine and estrogen [25]. Estrogen augments not only the differentiation of Treg cells [30] but also their suppressive activity through upregulation of programmed death (PD)-1 expression [31]. As the short-range *Chrna9* gene promoter was analyzed in breast cancer [25] and neuroblastoma [32] cell lines, the detailed regulatory mechanisms and functional role of α9-nAChR in iTreg cells and other T-cell subsets deserve further investigation.

In conclusion, TGF-β1 production, an important function of iTreg cells, was selectively suppressed by nicotine. Participation of α9-nAChR, regulated at the promoter activation level, in mediating nicotine’s effect on iTreg cells was suggested. They may be involved in the immunoregulatory role of the cholinergic system as well as neuro-immune interactions relevant to human health and diseases.

## 4. Materials and Methods

### 4.1. T-Cell Subset Preparation

The preparation of each T-cell subset was performed as previously described [10,11,17]. Briefly, six- to eight-week-old female BALB/c mice were obtained from Charles River Laboratories (Yokohama, Japan). Two to four mice employed in each set of experiments were allowed free access to water and food and were kept in a room at 23 ± 2 °C and 50 ± 10% humidity with a 12 h light/dark cycle. Naive CD4^+^ T cells were isolated from splenocytes using an EasySep Mouse Naive CD4^+^ T Cell Isolation Kit (Veritas, Tokyo, Japan). Cells were cultured in AIM-V medium (Thermo Fisher Scientific, Waltham, MA, USA) supplemented with 10% fetal bovine serum. At the start of culturing, pre-washed Dynabeads Mouse T-Activator CD3/CD28 (2 μL/10^5^ cells, Thermo Fisher Scientific) and recombinant IL-2 (10 U/mL, PeproTech, Inc., Cranbury, NJ, USA) were added. To promote differentiation of each subset, cells were cultured for 7 to 10 days in the presence of appropriate cytokines and anti-cytokine antibodies as previously described [10,11,17]. 

### 4.2. Cytokine Production and Proliferation of iTreg Cells

Differentiated iTreg cells were subjected to cytokine production and proliferation analyses. Cells were stimulated through CD3/CD28 as described above for 4 days in the presence or absence of nicotine. Proliferative responses and cytokine production in the culture supernatants were analyzed using the CellTiter 96 AQueous One Solution Cell Proliferation Assay (Promega, Madison, WI, USA) and enzyme-linked immunosorbent assay (ELISA) with mouse TGF-β1 and IL-10 DuoSet Kits (R&D Systems, Minneapolis, MN, USA), respectively, according to the manufacturers’ protocols.

### 4.3. Expression of nAChRs

The expression of nAChR messenger RNA (mRNA) in differentiated T-cell subsets and naive CD4^+^ T cells was analyzed by quantitative real-time reverse transcription PCR as previously described (16). Briefly, total RNA was extracted using an RNeasy Mini Kit (QIAGEN, Düsseldorf, Germany) and subjected to cDNA synthesis using Superscript VILO cDNA Synthesis Kit (Thermo Fisher Scientific) according to the manufacturer’s protocol. Then, 45 cycles of 2-step PCR (95 °C for denaturation and 60 °C for annealing and extension) were performed using Realtime PCR Master Mix (Toyobo, Osaka, Japan) and Assay-on-Demand Gene Expression Products (TaqMan minor groove binder probes; Thermo Fisher Scientific) for *Chrna2* (Mm00460630), *Chrna3* (Mm00520145), *Chrna4* (Mm00516561), *Chrna5* (Mm00616329), *Chrna6* (Mm00517529), *Chrna7* (Mm01312230), *Chrna9* (Mm01221611), *Chrna10* (Mm01274155), *Chrnb2* (Mm00515323), *Chrnb3* (Mm00532602), and *Chrnb4* (Mm00804952) with a StepOnePlus Real-Time PCR system (Thermo Fisher Scientific) and StepOne 2.3 Software (Thermo Fisher Scientific). The data are presented as relative mRNA abundance normalized to *Gapdh* (4352339E) expression as a control.

### 4.4. Chromatin Immunoprecipitation (ChIP) Assay

Histone modification at the *Chrna9* promoter region was analyzed by ChIP assay, as previously described [33]. Chromatin from the cells was enzymatically fragmented using a ChIP-IT Express Enzymatic Kit (Active Motif, Carlsbad, CA, USA) and was immunoprecipitated overnight at 4 °C with protein G magnetic beads and an antibody against tri-methylated lysine four in histone H3 (H3K4me3, Abcam, Cambridge, UK). The precipitated chromatin was eluted, reverse cross-linked, and treated with proteinase K according to the manufacturer’s instructions. Quantitative real-time PCR was performed using Power SYBR Green PCR Master Mix (Thermo Fisher Scientific). Primer sequences were as follows: *Chrna9* (Forward 5′-CTCTGCCTTCAAGCCAAATC-3′, reverse 5′-ACTGTGGCTCCAGTGACCTT-3′), *Hprt* (Forward 5′-CTCATGAGGAGGGAGAAAAATG-3′, reverse 5′-ATCTGACTAGGTGGGCCTGATA-3′). The data are presented as relative H3K4me3 levels normalized to the *Hprt* promoter region as a control.

### 4.5. Reporter Assay

*Chrna9* promoter-driven enhancer activity was assessed as previously described [34]. Briefly, the 5′-flanking sequence of the *Chrna9* gene (-10432 to -1) was cloned into the pEGFP-1 vector (Clontech Laboratories, Inc., Mountain View, CA, USA). The resulting construct was introduced into T cells by electroporation using a NEPA21 electroporator (Nepa Gene Co., Ltd., Chiba, Japan). Twenty-four hours after the transfection, the promoter activity was evaluated as the fluorescence of synthetically enhanced green fluorescent protein (EGFP) detected by flow cytometry. Human cytomegalovirus immediate early (CMV) promoter activity was also assessed by introducing the pCMV-DsRedEx vector (Clontech Laboratories, Inc.) as a control.

### 4.6. Statistics

All experimental data are presented as means ± standard deviation (SD). Statistical analyses were performed using Student’s *t*-test or one-way analysis of variance with Dunnett’s test. Statistical significance was set at *p* < 0.05.
